# MicroRNA-155 influences cell damage in ischemic stroke via TLR4/MYD88 signaling pathway

**DOI:** 10.1080/21655979.2021.1935066

**Published:** 2021-06-28

**Authors:** Wei Chen, Lingtong Wang, Zhaoping Liu

**Affiliations:** a Department of Neurology, Linhai Second People’s Hospital, Taizhou, 317016, Zhejiang, China; b Department of rehabilitation medicine, Chenzhou first people’s Hospital (the First Affiliated Hospital of Xiangnan University), Chenzhou, 423000, Hunan, PR China

**Keywords:** MiR-155, tlr4/myd88, ischemic stroke, cell damage

## Abstract

Cerebral ischemic stroke (CIS) is extremely harmful, and its treatment should be underpinned by understanding its pathogenic mechanism. This study was designed to determine the involvement of miR-155 in CIS development via the TLR4/MyD88 signaling pathway. First, we quantified serum miR-155 in patients with CIS and healthy individuals, and found high expression of miR-155 in such patients and a decrease in it in the patients after therapy (*P* < 0.05). Serum miR-155 demonstrated a favorable function in predicting the development and prognosis of CIS (*P* < 0.001). We also conducted a mouse assay, and found that knocking out miR-155 can improve the neurological function of mice and suppress protein TLR4 and MyD88 (all *P* < 0.05). Finally, we carried out a cell assay, and found enhancement in the activity of SH-SY5Y cells, decrease in their apoptosis, and protein TLR4 and MyD88 in them after suppression of miR-155 (all *P* < 0.05). Furthermore, we also found complete reverse by TLR4/MyD88 pathway inhibitor on the influence of increasing miR-155 on cells (*P* > 0.05). Therefore, with an increase in cases with CIS, miR-155 takes a part in the development of cell damage by activating TLR4/MyD88, and it is probably the key to diagnosing and treating CIS.

## Graphical abstract

**Figure:**
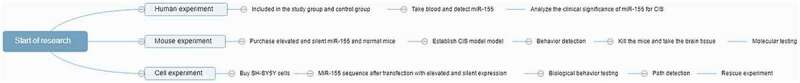

## 1. Introduction

Cerebral ischemic stroke (CIS), also known as cerebral infarction, is a situation of brain tissue necrosis due to insufficiency of cerebral blood supply caused by dysfunction of supplying arteries [1]. With an extremely high global incidence, CIS is prevalent in middle-aged and senior individuals [2]. According to statistics, there are over 8 million new patients with CIS each year, and the number is increasing annually [3,4]. CIS can be manifested as pain, numbness and weakness of limbs, dysfunction of consciousness and language, paralysis, and it may even result in sudden death in severe cases [5], taking a heavy toll on patients. Currently, CIS is primarily treated by favored conservative therapy, but due to its fast and sudden development, conservative therapy can only achieve remission and is unable to completely cure it [6,7]. For some patients with it under serious situation, only surgical treatment is selectable, but the prognosis of patients after surgery is not optimistic [8]. Dysfunction of cerebral supplying arteries is known as the cause of CIS, but its definite development remains unclear [9]. Therefore, domestic and foreign researchers are working hard to probe into the mechanism of CIS molecularly to find a novel and effective therapy scheme.

As research deepens, increasing researchers point out the possible close relationship between CIS development and micro RNA, such as involvement of miR-126 in CIS and great significance of miR-146a in coronary heart disease [10,11]. Among the various miRNAs, miR-155 is associated with a variety of different neurodegenerative diseases [12]. And miR-155 shows high expression in brain injury diseases such as cerebral ischemia and glioma [13], and has been found to regulate the activity of vascular endothelial cells (VECs) [14]. The development of CIS is closely related to the accelerated apoptosis of vascular endothelial cells [15]. Therefore, we conjectured that miR-155 may affect CIS development by regulating the blood supply capacity of blood vessels, but no earlier study has verified our conjecture. Moreover, we also learnt that TLR4/MyD88 is highly crucial in brain diseases, and is also able to regulate the function of VECs [16]. Zhu et al. found that diosin suppressed CIS-induced inflammatory responses by inhibiting the TLR4/MyD88/NF -κB signaling pathway [17]. Additionally, miR-155 has been discovered to be a part in kidney injury via activation of TLR4/MyD88 signaling pathway [18].

Therefore, we further speculated that the involvement of miR-155 in CIS was also related to the pathway. CIS poses increasingly severe clinical challenges. Fully understanding the mechanism of miR-155 in it is probably the breakthrough of diagnosing and treating CIS. Thus, this study probed into the influence mechanism of miR-155 in CIS and its relationship with TLR4/MyD88 to provide a reliable theoretical basis for treating patient with CIS.

## 2. Materials and methods

### 2.1 Patients

Patients with CIS admitted to the neurology department of Linhai Second People’s Hospital between May 2017 and June 2019 were enrolled into a research group (res group). According to the inclusion criteria (patients diagnosed as CIS via head imaging, patients between 40 and 60 years old, and those without other comorbid diseases), 96 patients were enrolled. Then, according to the exclusion criteria (patients with organ dysfunction, patients transferred midway, and those having long taking antibiotics), 61 patients were finally enrolled. Additionally, healthy individuals were enrolled into a control group (con group). Based on the inclusion and exclusion criteria (patients having not suffered major diseases and those between 40 and 60 years old), 73 healthy individuals were enrolled finally. Patients in the res group were followed up via reexamination, and their diseases recurrence was recorded. This experiment was conducted with approval from the Ethics Committee of our hospital, and all enrolled participants signed informed consent forms.

### 2.2 Sample collection

After admission, patients with CIS were treated in strict accordance with standardized treatment guidelines for CIS [19]. Fasting venous blood (4 mL) sampled from each patient at admission and discharge and from each healthy individual were transferred to coagulation-promoting tubes, followed by centrifugation (300 × g, 4°C) for collecting serum, and the collected serum was stored in a refrigerator (−80°C) for later analyses.

### 2.3 Animals and model

Thirty C57BL mice (6–8 weeks old, half male and half female) were purchased from Beijing Institute of Brain Science and Brain-Inspired Intelligence, with animal license number of SYXK (Beijing) 2019–0029. All mice were randomly divided into three groups: 10 mice with miR-155 overexpression (miR-155 OE group), 10 mice with miR-155 low expression (miR-155 Low group) and 10 normal mice (Control group).

Each mouse of the groups was injected intraperitoneally with 10% chloral hydrate (3 mL/kg) for anesthesia, followed by cutting of the skin of the left neck and blunt dissection of surrounding muscle tissues to dissociate associated cervical arteries, and then the proximal end of the common carotid artery and the external carotid artery were ligated, and the distal end of the internal carotid artery was clamped. A fishing line was inserted into the site 1 cm away from the common carotid artery, and the stump was fastened and sutured once obvious resistance occurred Afterward, the neurological function of the mouse was scored [20], and a score between 3 and 4 points indicated successful modeling. On the 8^th^ day, all mice were executed by cervical dislocation under anesthesia, and their left hippocampal tissues were obtained.

### 2.4 Morris water maze test

The water maze was 120 × 60 cm in size and 25 cm deep (20 ֯C), divided into four quadrants, with a 10 × 23 cm diameter platform placed in the middle of the third quadrant. And the mice were randomly placed into the maze and guided in finding the platform. The mice were trained twice a day, and the time of each mouse for finding the platform (escape latency, EL) without guidance within 6 s on the 6^th^ day was recorded.

### 2.5 Cells and cell transfection

SH-SY5Y cells purchased from the ATCC were subjected to 12 h incubation in 95%N_2_ under hypoxia in medium with cell oxygen and glucose deprivation (pH 7.2–7.4) that was then changed to DMEM supplemented with 10% fetal bovine serum (FBS) for subculture. MiR-155-mimics, miR-155-inhibition, and miR-155-NC were constructed, followed by transfection to cells in 2.5 via Lipofectamine™ 2000 (Invitrogen, USA).

### 2.6 Nano-PCR for miR-155 quantification

Nano-magnetic beads purchased from Thermo Fisher Scientific, USA were placed on the glass slide and physiological saline was dropped on them. Then the morphology of nano-magnetic beads was evaluated by TEM. Subsequently, reverse transcription was conducted to total RAN from nano-magnetic beads to obtain cDNA that was then subjected to amplification, followed by PCR detection. With U6 as internal reference, 2^−ΔΔCt^ was used for expression calculation (Table 1 for primer sequences). This experiment was repeated three times.

**Table 1.** Primer sequences

Gene	Forward (5'-3')	Reverse (5'-3')
MiR-155	GCCGCTTAATGCTAATCGTG	CAGTGCTGGGTCCGACTGA
U6	CTCGCTTCGGCAGCACA	AACGCTTCACGAATTTGCGT

### 2.7 CCK-8 assay

Transfected cells were subjected to 48 h incubation, followed by addition of 10 *μ*L CCK-8 per well and 2 h incubation with dark surroundings. Finally, the cells were determined via a microplate reader (450 nm). This experiment was repeated three times.

### 2.8 Flow cytometry

Transfected cells were trypsinized via 0.25% trypsin, followed by addition of binding buffer for preparing 1*10^6^个/mL cell suspension that was successively added with AnnexinV-FITC and PI (5 *μ*L). Afterward, the cells were subjected to 5-min incubation with dark surroundings, and then analyzed by a FC500MCL flow cytometer. This experiment was repeated three times.

### 2.9 Western blot

Total protein lysates were separated from the brain tissues or SH-SY5Y cells by 10% SDS-PAGE, and then transferred to polyvinylidene fluoride (PVDF) membranes. After blocked with 5% skimmed milk for 1 h at room temperature, the membranes were incubated with primary antibodies (TLR4, MyD88, and GAPDH) at 4°C overnight, followed by incubation with the secondary antibody for 2 h at room temperature. The membranes were visualized by ECL regents. This experiment was repeated three times.

### 2.10 Statistical analyses

Results recorded as the mean ± SD were analyzed via SPSS22.0. Inter-group comparison and comparison of data before and after therapy were carried out via the independent-samples t-test and paired t-test, respectively, and multi-group comparison of data was carried out using the one-way ANOVA followed by LSD t-test. *P* < 0.05 denotes a notable significance.

## 3. Results

### 3.1 MiR-155 expression

**Figure 1.**
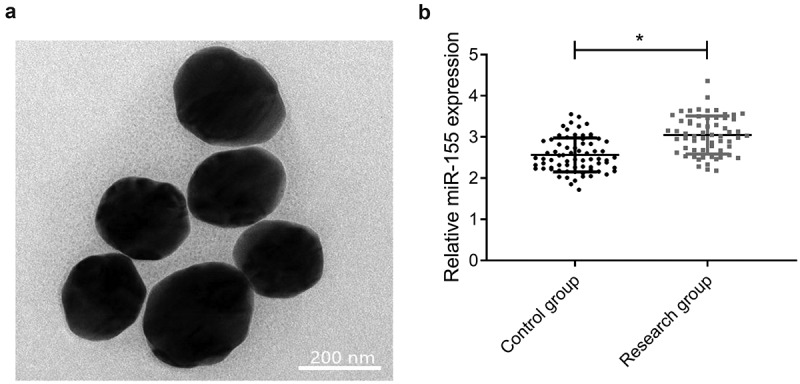
MiR-155 was up-regulated in serum of patients with CIS. (a) Nano-magnetic beads under TEM. (b) MiR-155 expression was detected by Nano-PCR. *P < 0.05

First, we determined the expression of miR-155 in CIS patients by Nano-PCR. According to results by TEM, nano-magnetic beads were round with uniform size, smooth surface and tight arrangement (Figure 1A). The res group showed higher miR-155 expression than the con group (3.08 ± 0.46 *vs*. 2.56 ± 0.41, *P* < 0.05) (Figure 1B). This result suggests that miR-155 was highly expressed in the serum of CIS patients and may be involved in the progression of CIS.

### 3.2 Clinical significance of miR-155

**Figure 2.**
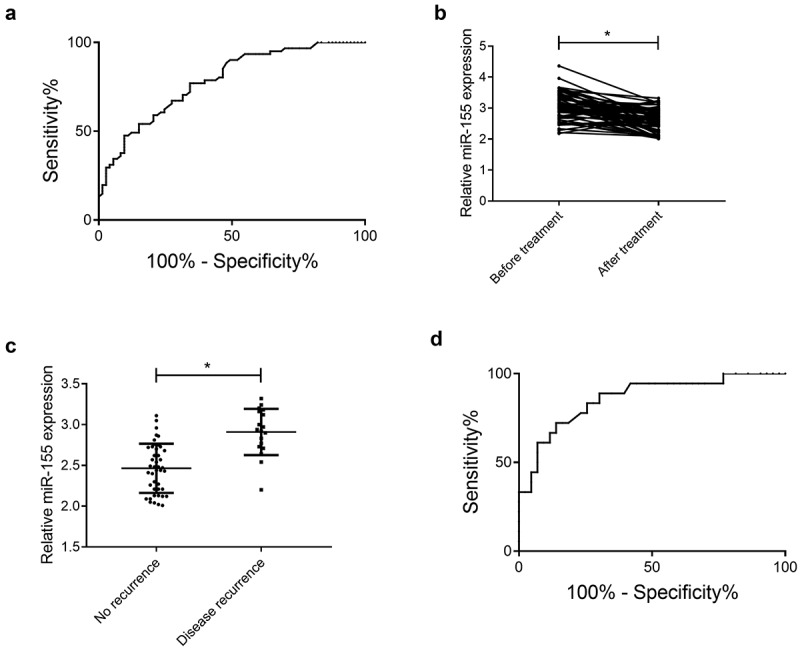
Clinical significance of miR-155. (a) Prediction of CIS development via miR-155 according to ROC analysis, AUC: 0.783, 95% CI: 0.707–0.859. (b) MiR-155 before and after therapy, *P < 0.05. (c) MiR-155 in patients with recurrence and those without it, ***P < 0.001. (d) Prediction of CIS recurrence via miR-155 according to ROC analysis, AUC: 0.861, 95% CI: 0.758–0.965

According to results of ROC, miR-155 > 2.665 had a sensitivity and specificity of 77.05% and 65.75% in predicting CIS development (*P* < 0.001, Figure 2A). After therapy, miR-155 decreased (2.60 ± 0.36) (*P* < 0.001, Figure 2B). Additionally, follow-up results of 18 patients with recurrence revealed that patients with recurrence had higher miR-155 expression than those without it (*P* < 0.001, Figure 2C), and after therapy, miR-155 > 2.630 had a sensitivity and specificity of 88.89% and 69.77% in predicting CIS recurrence (*P* < 0.05, Figure 2D).

### 3.3 Results of water maze test

**Figure 3.**
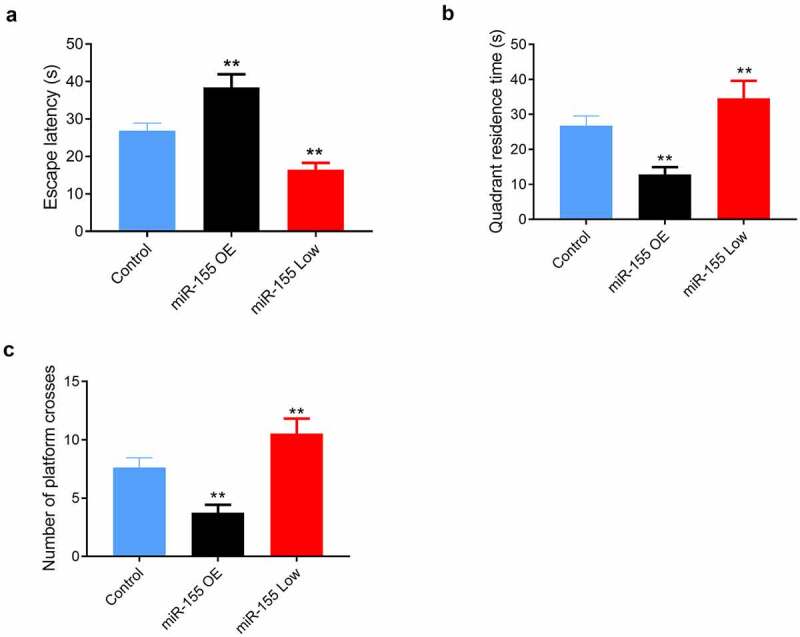
Effects of miR-155 on spatial learning and memory in mice in the Morris water maze test. (a) Mean escape latency of reaching the submerged platform in the training period. (b) Quadrant residence time. (c) Number of platform-site crossover. 10 mice in each group. MiR-155 OE, miR-155 overexpression. **P < 0.01 vs. Control group

Further, we determined the effect of miR-155 expression changes on the neurological function of CIS mice by *in vivo* experiments. The EL, quadrant residence time (QRT), and number of platform-site crossover (PSC) of miR-155 OE group were (38.42 ± 3.52 s), (12.84 ± 2.08 s) and (3.75 ± 0.68 times), respectively, those of miR-155 Low group were (16.42 ± 1.84 s), (34.63 ± 4.96 s), and (10.54 ± 1.28 times), respectively, and those of Control group were (26.87 ± 2.04 s), (26.72 ± 2.84 s), and (7.63 ± 0.84 times), respectively. miR-155 OE group showed longest EL and least QRT and number of PSC (all *P* < 0.05, Figure 3A, B, C), and miR-155 Low group showed shorter EL and more QRT and number of PSC than Control group (both *P* < 0.05, Figure 3A, B, C).

### 3.4 MiR-155, TLR4, and MyD88 in mouse brain tissues

**Figure 4.**
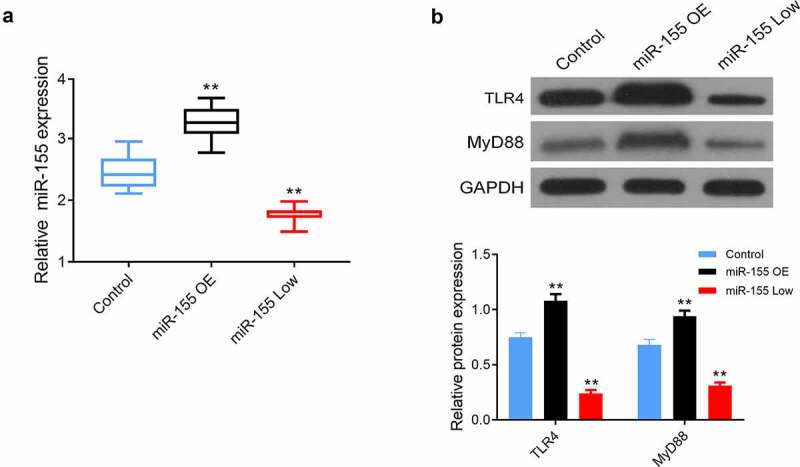
Effects of miR-155 on TLR4/MyD88 signaling pathway in mouse brain tissues. (a) The expression of miR-155 in brain tissues was detected by Nano-PCR. (b) The protein expression of TLR4 and MyD88 in brain tissues was measured by western blot. 10 mice in each group. MiR-155 OE, miR-155 overexpression. **P < 0.01 vs. Control group

In addition, miR-155 in the brain tissues of miR-155 OE group, miR-155 Low group, and Control group was 3.27 ± 0.29, 1.77 ± 0.13, and 2.46 ± 0.27, respectively, and the highest miR-155, TLR4, and MyD88 were found in miR-155 OE group, and the levels of them in miR-155 Low group were lower than those in Control group (all *P* < 0.05, Figure 4A, B).

### 3.5 Influence of miR-155 on SH-SY5Y

**Figure 5.**
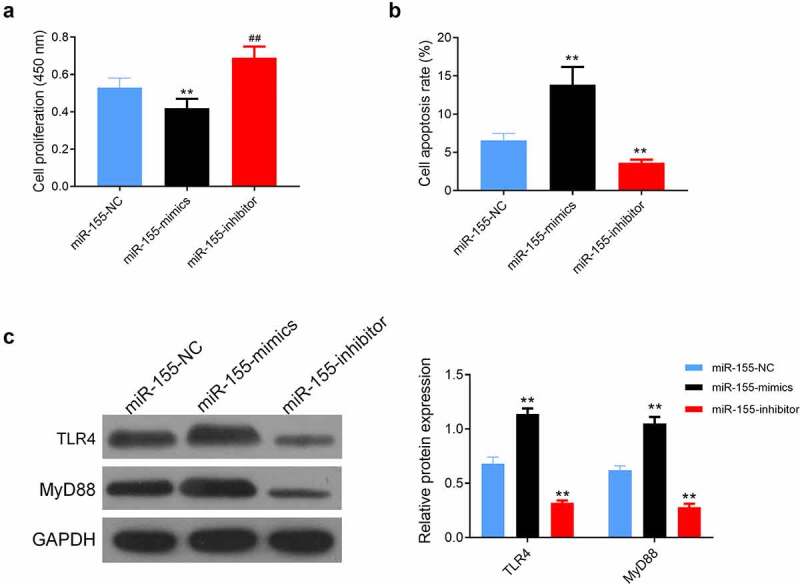
Effects of miR-155 on SH-SY5Y cell. (a) Cell proliferation was detected by CCK-8 assay. (b) Flow cytometry was used to measure cell apoptosis. (c) The protein expression of TLR4 and MyD88 in cells was evaluated by western blot. **P < 0.01 vs. miR-155-NC group

*In vitro* assay verified lower cell proliferation and higher apoptosis in the miR-155-mimics group than in the miR-155-inhibitor and miR-155-NC groups (both *P* < 0.05, Figure 5A, B), and higher proliferation and lower apoptosis in the miR-155-inhibitor group than those in the miR-155-NC group (*P* < 0.05, Figure 5A, B), highest TLR4 and MyD88 expression in the miR-155-mimics group, and lower TLR4 and MyD88 expression in the miR-155-inhibitior group than that in the miR-155-NC group (*P* < 0.05, Figure 5C).

### 3.6 Impact of miR-155 on SH-SY5Y via TLR4/MyD88

**Figure 6.**
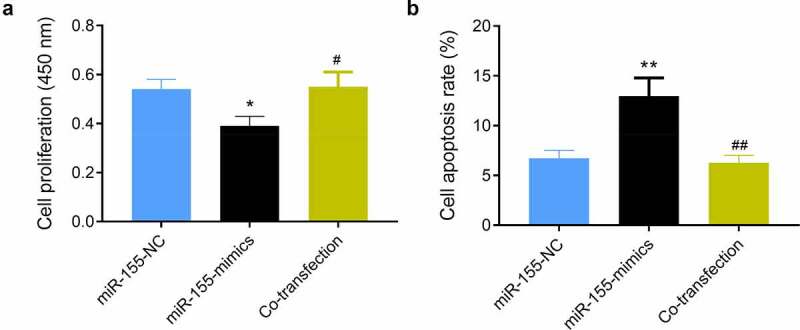
Impact of miR-155 on SH-SY5Y via TLR4/MyD88. (a) Cell proliferation was detected by CCK-8 assay. (b) Cell apoptosis was determined using flow cytometry. *P < 0.05 and **P < 0.01 vs. miR-155-NC group; ^#^*P* < 0.05 and ^##^*P* < 0.01 vs. miR-155-mimics group. Co-transfection group, miR-155-mimics and TJ-M2010-2

SH-SY5Y cells co-transfected with miR-155-mimics and TJ-M2010-2 (co-transfection group) showed consistent proliferation and apoptosis with those transfected with miR-155-NC (both *P* > 0.05, Figure 6A, B), and also showed higher proliferation and lower apoptosis than the miR-155-mimics group (*P* < 0.05, Figure 6A, B).

## 4. Discussion

CIS is fatal, and understanding its pathogenic mechanism is of great significance [21]. This study preliminarily analyzed the role of miR-155 in CIS based on human specimens and animal models and cells. Despite various many limitations, this can lay a solid foundation for the future molecular pathogenesis of CIS. We conjectured that monitoring and observing miR-155 in the future can enable us to timely diagnose CIS development and evaluate its progression, and probably reveal the potential of miR-155 as a therapeutic target.

This study firstly quantified serum miR-155 in patients with CIS and healthy individuals, finding high expression in the former. The results preliminarily suggest that miR-155 probably takes a part in CIS development. Earlier studies have also found its high expression in cases with fatty liver or chronic obstructive pulmonary disease [22,23], which can also testify our experimental results. For investigating the clinical value of miR-155 in CIS, we analyzed the predictive value of it to CIS through ROC curve, and found its favorable effect in predicting CIS development (sensitivity = 77.05%, specificity = 65.75%). CIS is mainly diagnosed via imaging currently, and its results are mainly evaluated based on the experience and subjective opinions of clinicians. Sometimes, imaging is unable to accurately display CIS with complicated lesions, so imaging is accompanied by a certain risk of missed diagnosis and misdiagnosis [24]. Serum markers are both convenient and efficient, and their quantification is extremely objective. Analysis of ROC needs more data about patients, but the number of patients enrolled in our study is relatively small, so we are unable to obtain the most accurate results. We will address it as soon as possible. Then, we compared the changes of miR-155 in patients after therapy, and found a notable decrease in it after therapy, which further verified that miR-155 participated in CIS development and changed with the changes of the disease. Based on follow-up for prognosis, we found higher miR-155 expression in patients with recurrence than in those without it, and its favorable predictive value for the prognosis and recurrence of patients. The results further verified our above conjecture again, and also simply confirmed the clinical value of miR-155 in CIS. We believe that based on improved clinical study, quantification of miR-155 will enable us to not only judge CIS development, but also understand the development and prognosis of the disease, which is convenient for timely clinical intervention and improves patients’ prognosis.

Subsequently, for further analyzing the impact of miR-155 on CIS, we constructed CIS models through mice with overexpressed miR-155, we found that miR-155 overexpression mice had significantly poorer learning and memory functions than the normal group, while miR-155 knockout mice had significantly enhanced learning and memory abilities. During CIS development, due to the dysfunction of cerebrovascular blood supply function, brain tissues have different degrees of hypoxia injury and necrosis, and cause a certain degree of neurothinking dysfunction [25,26]. The results of this experiment preliminarily suggest that silencing miR-155 can improve the neural function of mice with CIS and reduce the damage of brain tissue. Furthermore, we also found highest TLR4 and MyD88 protein in miR-155 overexpression group. The results denote abnormal expression of TLR4/MyD88 in cases with CIS. TLR4, a transmembrane protein, can reveal various pathogen-associated molecular structures, give rise to immune response, and thus destroy the normal morphology of the original environment [27]. MyD88 medicating the production and release of inflammatory mediators as a downstream transfection factor of TLR4, is the key substance of inflammatory injury [28]. TLR4/MyD88 has been verified to be involved in tumor diseases and be bound up with damage of cerebrovascular and brain tissues [29–31], and the above experiments also preliminarily confirmed it. Finally, we constructed injured cells with CIS by oxygen and sugar deprivation, and performed CCK-8 and flow cytometry assays. We found enhancement in the activity of SH-SY5Y cells, decrease in their apoptosis and protein TLR4 and MyD88 in them after suppression of miR-155, and also found reverse results after up regulation of it. The results imply that miR-155 can damage cells by activating TLR4/MyD88. Through the rescue experiment, we also found complete reverse by TLR4/MyD88 pathway inhibitor on the influence of increasing miR-155 on cells, which confirmed the regulatory relationship between them. Based on the above, we can confirm the participation of miR-155 in CIS development by activating TLR4/MyD88.

However, CIS development is a complex process of multi-gene and multi-channel influence, which has been confirmed in many previous studies [32,33]. Therefore, the mechanism of miR-155 participating in CIS is worthy of further study and discussion. Because of the short experimental period, we are unable to evaluate the impact of miR-155 on the long-term prognosis of patients with CIS. We will conduct corresponding supplementary experiments as soon as possible to improve our study.

## Conclusion

In summary, we found that miR-155 is elevated in CIS and is involved in the development of CIS by activating the TLR4/MyD88 signaling pathway causing cellular injury. This regulatory pathway provides new insights into the mechanisms underlying the progression and development of CIS and may be key to the diagnosis and treatment of CIS in the future.

## Highlight

1. MiR-155 was upregulated in patients with cerebral ischemic stroke (CIS).
2. Spatial learning memory was significantly reduced in mice with high miR-155 expression.
3. MiR-155 causes SH-SY5Y cell injury through activation of TLR4/MyD88 signaling pathway.
